# Multi-label multi-instance transfer learning for simultaneous reconstruction and cross-talk modeling of multiple human signaling pathways

**DOI:** 10.1186/s12859-015-0841-4

**Published:** 2015-12-30

**Authors:** Suyu Mei, Hao Zhu

**Affiliations:** 1 Software College, Shenyang Normal University, Shenyang, China; 2 Bioinformatics Section, School of Basic Medical Sciences, Southern Medical University, Guangzhou, China

## Abstract

**Background:**

Signaling pathways play important roles in the life processes of cell growth, cell apoptosis and organism development. At present the signal transduction networks are far from complete. As an effective complement to experimental methods, computational modeling is suited to rapidly reconstruct the signaling pathways at low cost. To our knowledge, the existing computational methods seldom simultaneously exploit more than three signaling pathways into one predictive model for the discovery of novel signaling components and the cross-talk modeling between signaling pathways.

**Results:**

In this work, we propose a multi-label multi-instance transfer learning method to simultaneously reconstruct 27 human signaling pathways and model their cross-talks. Computational results show that the proposed method demonstrates satisfactory multi-label learning performance and rational proteome-wide predictions. Some predicted signaling components or pathway targeted proteins have been validated by recent literature. The predicted signaling components are further linked to pathways using the experimentally derived PPIs (protein-protein interactions) to reconstruct the human signaling pathways. Thus the map of the cross-talks via common signaling components and common signaling PPIs is conveniently inferred to provide valuable insights into the regulatory and cooperative relationships between signaling pathways. Lastly, gene ontology enrichment analysis is conducted to gain statistical knowledge about the reconstructed human signaling pathways.

**Conclusions:**

Multi-label learning framework has been demonstrated effective in this work to model the phenomena that a signaling protein belongs to more than one signaling pathway. As results, novel signaling components and pathways targeted proteins are predicted to simultaneously reconstruct multiple human signaling pathways and the static map of their cross-talks for further biomedical research.

**Electronic supplementary material:**

The online version of this article (doi:10.1186/s12859-015-0841-4) contains supplementary material, which is available to authorized users.

## Background

Signaling pathways play important roles in the life processes of cell growth, differentiation and apoptosis. The stimuli from extracellular environment and cellular matrix are sensed, amplified and transducted to nucleus via signaling pathways to yield complex biological responses (e.g. enzyme activity, transcription factors activation/deactivation, gene expression, ion-channel activity, etc.) [1]. Malfunction of signaling pathways is likely to lead to a variety of pathologies [2].

Protein-protein interaction (PPI) networks play fundamental roles in the study of signaling transduction, because extracellular signals are generally transmitted from membrane to nucleus via a series of PPIs and molecular modifications. Thus reconstruction of PPI networks, including experimental techniques [2, 3] and computational modeling [4–8], has attracted much attention in recent years. At present, the existing computational methods for reconstruction of signaling pathways mainly rely on shortest path algorithm [9–11] and message-passing algorithm [12]. For instance, Tuncbag et al. [12] used message-passing algorithm to derive directed forest from PPI networks, based on which to infer signaling pathways. These methods are simple with least demanding data constraint in that only PPI network topology is needed. Besides, the method [12] used confidence weighted interactome to explicitly counteract the noise of PPI network topology, so that the risk of false negatives and false positives is reduced. Nevertheless, PPI network topology based methods need to be further improved from the two concerns: (1) signaling pathways possibly contain feedback loops that make the shortest path algorithm inaccurate to yield false signaling components; (2) the experimental data of signaling components should be exploited to guide the search of novel signaling components in PPI networks.

Comparatively machine learning methods are effective to simultaneously exploit multiple experimental data of signaling pathways without prior knowledge about the underlying biochemical mechanism [13–16]. Reconstruction of signaling pathways can be decomposed into two steps: the first step is recognition of signaling components, and the second step is to link the predicted signaling components to the existing signaling pathways via experimental PPIs or predicted PPIs. The existing machine learning methods focus on the discovery of novel signaling components [14–16]. In [14], a multi-class SVM model is trained using the feature information of protein domain to predict novel signaling components. In [15], the data of experimentally verified signaling components are used to train a SVM model for the prediction of homologous signaling pathways. In [16], the ortholog pairs of known interacting signaling components from known signaling pathways, called signalogs, are directly treated as signaling PPIs and then used the signalogs to construct homologous signaling pathways. Actually, computational reconstruction of signaling pathways, as a complex problem, needs to address the three major concerns: (1) discovery of novel signaling components, especially those signaling components that belong to more than one signaling pathway; (2) linking to signaling pathways the predicted signaling components via signaling PPIs; (3) cross-talk modeling between signaling pathways. To our knowledge, the existing computational methods seldom explicitly address the three major concerns to date. Recently, cross-talk modeling between signaling pathways has aroused much attention. For instances, graph search method is used to find the common cross-talk signaling components between the three signaling pathways (EGFR, IGF-1R and IR)[17], and PRISM modeling language is used to formally describe the common modules between signaling pathways [18]. Unfortunately, these methods are generally descriptive and can neither predict novel signaling components nor model signaling cross-talks.

In this work, we propose a multi-label multi-instance transfer learning method to simultaneously reconstruct multiple human signaling pathways and model their cross-talks. In this method, the data of the known signaling components from 27 human signaling pathways are used to train a 28-class multi-label SVM (support vector machine) model, wherein the 28^th^ class contains the negative data that are randomly sampled from the proteins that do not belong to the 27 signaling pathways. The scenario that a signaling component is shared by or belongs to multiple signaling pathways is modeled under multi-label learning framework. To enrich the knowledge of the protein concerned, each protein is depicted with two instances, one instance called target instance is represented with its own gene ontology annotations, and the other instance called homolog instance is represented with the gene ontology annotations of its homologs. Besides enriching the target instance, the homolog instance is especially useful to substitute the target instance when the protein concerned is completely not annotated. Unlike traditional supervised learning, the evaluation of multi-label learning model is conducted using three performance metrics, namely exact match ratio, microaverage F-measure and macroaverage F-measure. To evaluate the reliability of the proposed model, we validate the proteome-wide predictions against recent literature as well as conduct cross validation on the training data. Then we link the predicted signaling components to signaling pathways via experimental PPIs and derive the cross-talks between the 27 human signaling pathways to provide valuable cues for further biomedical research.

## Data and methods

### Human signaling pathways

To date there are several major signaling pathway databases for free academic use, e.g. KEGG (Kyoto Encyclopedia of Genes and Genomes) [19], Reactome [20], SPAD (Signaling Pathway Database) [21], NetPath [22], SignaLink [23] etc. In this work, we choose NetPath (http://www.netpath.org/) to construct the training for the reasons: (1) NetPath manually curates 35 human cancer/immune signaling pathways, the largest repository of human cancer signaling pathways at present to our knowledge; (2) The signaling components explicitly provided by NetPath are conveniently treated as the training data. KEGG is rather small and contains a limited number of human signaling pathways. The other databases like Reactome and SignaLink are timely updated, but likewise collect very limited number of human cancer signaling pathways so as not to directly serve our purpose. We incorporate the closely related cancerogenic signaling pathways into a single model to facilitate effective knowledge sharing. To date NetPath has manually curates 35 human immune/cancer signaling pathways that contain 11 sub-types of Interleukin (IL-1 ~ IL-11). For simplicity, IL-1 ~ IL-11 are merged into one single class, thus we obtain 27 human signaling pathways as shown in Table 1. The signaling components provided on the website (http://www.netpath.org/) are directly used as training data and the training data are further validated against SwissProt database [24] and GOA database [25] to remove those proteins that are not manually curated and contain empty set of gene ontology annotations. The number of signaling components of each signaling pathway is shown in Table 1.

**Table 1:** Statistics of the predicted signaling components and the derived signaling PPIs for the 27 human signaling pathways

Class name	Name of signaling pathway	Size	#Novel SC			#Novel signaling PPI		
			TI	HI	∩	TI	HI	∩
Notch	Notch receptor	83	67	239	27	56	126	25
TCR	T cell receptor	260	660	779	221	418	431	125
TGFBeta	Transforming growth factor beta receptor	216	293	563	94	336	545	104
TNF	Tumor necrosis factor alpha	318	653	1016	302	493	985	261
Wnt	Wnt signaling	108	92	219	49	36	89	23
IL	Interleukin (IL-1 ~ IL-11)	260	131	502	65	167	394	55
Alpha6	Alpha6 beta4 integrin	74	36	158	1	2	83	0
AR	Androgen receptor	173	649	495	156	535	321	104
BCR	B cell receptor	175	177	298	52	96	160	29
BDNF	Brain-derived neurotrophic factor	128	139	373	64	106	212	65
CRH	Corticotropin-releasing hormone	71	27	124	3	28	43	4
EGFR	Epidermal growth factor receptor	432	104	1206	426	110	118	446
FGF1	Fibroblast growth factor-1	103	105	202	15	34	120	1
FSH	Follicle-stimulating hormone	53	25	145	14	3	36	1
Gastrin	Gastrin signaling	94	10	196	3	2	91	2
Ghrelin	Ghrelin receptor	76	45	213	27	32	103	24
Hedgehog	Hedgehog signaling	36	19	70	11	34	19	2
ID	Inhibitor of differentiation	45	41	79	13	26	19	9
Kit	Kit receptor	110	4	128	3	7	89	4
Leptin	Leptin signaling	107	7	179	4	8	103	8
OSM	Oncostatin-M	77	1	120	0	1	54	0
Prolactin	Prolactin receptor	115	23	222	7	14	113	4
RAGE	Advanced glycation end-products	92	5	195	2	10	84	0
RANKL	Receptor activator of nuclear factor kappa-B ligand	85	92	147	4	16	56	3
TSH	Thyroid-stimulating hormone	105	33	229	20	26	91	14
TSLP	Thymic stromal lymphopoietin	192	797	463	129	186	132	8
TWEAK	TNF-related weak inducer of apoptosis	46	5	91	1	6	38	0
Others	Other class or miscellaneous proteins	432						

#Novel SC denotes the number of predicted novel signaling components. TI denotes the target instance case, HI denotes the homolog instance and ∩denotes the intersection

**Fig. 1:**
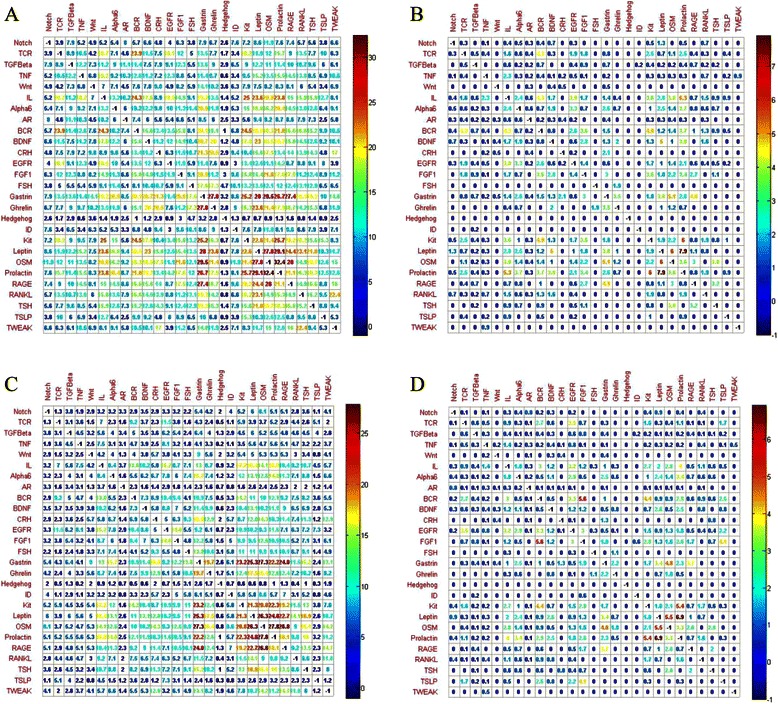
**a** Matrix plots cross-talk ratio (%) of signaling components between the experimental human signaling pathways. **b** Matrix plots the cross-talk ratio (%) of signaling PPIs between the experimental human signaling pathways. **c** Matrix plots cross-talk ratio (%) of signaling components between the reconstructed human signaling pathways. **d** Matrix plots the cross-talk ratio (%) of signaling PPIs between the reconstructed human signaling pathways. The values along the diagonals are trivial and the color bar is used to highlight the magnitude of cross-talks between two human signaling pathways

In general, signaling pathways temporally and spatially communicate via common signaling components and common signaling PPIs. Take the experimental NetPath database for example, EGFR signaling pathway shares 108 common signaling components with Interleukin signaling pathway and 106 common signaling components with TCR signaling pathway. To measure the relatedness of any two signaling pathways, we define two cross-talk ratios: the cross-talk ratio of signaling components (*CTR*_*SC*_) and the cross-talk ratio of signaling PPIs (*CTR*_*SPPI*_). Assume *A*_*SC*_ and *B*_*SC*_ to denote the sets of signaling components of two signaling pathway *A* and *B*, then *CTR*_*SC*_ is defined as *CTR*_*SC*_ = |*A*_*SC*_ ∩ *B*_*SC*_|/|*A*_*SC*_ ∪ *B*_*SC*_|, where |*A*| denotes the cardinality of set *A. CTR*_*SC*_ is actually the ratio of the overlap between set *A*_*SC*_ and set *B*_*SC*_. The cross-talk ratio of signaling components (*CTR*_*SC*_)that is derived from the experimental NetPath database is illustrated in Fig. 1(a). We see that there generally are a certain number of signaling components shared between any two signaling pathways. Take TCR for instance, TCR seems to be more correlated with IL (*CTR*_*SC*_ = 18.7 %), BCR (*CTR*_*SC*_ = 23.9 %), EGFR (*CTR*_*SC*_ = 18.1 %) and Kit (*CTR*_*SC*_ = 18.2 %).

Similarly assume *A*_*SPPI*_ and *B*_*SPPI*_ to denote the sets of signaling PPIs of two signaling pathway *A* and *B*, we define the cross-talk ratio of signaling PPIs as *CTR*_*SPPI*_ = |*A*_*SPPI*_ ∩ *B*_*SPPI*_|/|*A*_*SPPI*_ ∪ *B*_*SPPI*_| and derive the cross-talk ratio *CTR*_*SPPI*_ from the experimental NetPath database as illustrated in Fig. 1(b). Comparatively, the cross-talk ratio *CTR*_*SPPI*_ is generally much lower than the cross-talk ratio *CTR*_*SC*_, implying that a signaling pathway more depends on cross-talk signaling components to stimulate or communicate with other signaling pathways than cross-talk signaling PPIs. Take TCR for example again, TCR seems to be more correlated with BCR (*CTR*_*SPPI*_ = 4.3 %).

Using the obtained signaling components, we can easily train a predictive model to predict an unseen protein to one or more than one signaling pathway. To handle the case that a protein may not belong to any of the 27 signaling pathways, we need to construct a negative class for the completeness of classification. The negative class named *others* contains the proteins that either are not signaling proteins or do not belong to the 27 signaling pathways. The data of the *others* are randomly sampled from the proteins that do not belong to the 27 signaling pathways. Actually the space of class *others* is very large, we restrict the size of class *others* equal to that of the class that contains maximum number of signaling components for the purpose of reducing the risk of model bias.

### Multi-label multi-instance transfer learning

#### Transfer learning

Transfer learning has been proven effective in knowledge/ information transfer across related but heterogeneous domains [26]. In recent years, transfer learning, sometimes in the form of multi-task learning, has found many applications in computational biology [4, 5, 27–30]. Knowledge transfer is generally conducted via model parameter optimization [27] and evolutionary homologs [4, 5, 28–30]. As compared with the methods of object function optimization, homolog knowledge transfer is easy to be biologically interpreted and is robust against data unavailability. The machine learning frameworks that are adopted to implement knowledge transfer include ensemble learning [4, 30], multi-instance learning [5], semi-supervised learning [27] and multi-kernel learning [28, 29].

In this work, we use multi-learning framework to model the phenomena that a signaling protein belongs to more than one signaling pathway, and use multi-instance learning framework to implement homolog knowledge transfer. Each protein is represented with two instances, one instance called target instance is used to represent the GO feature information of the protein itself, and the other instance called homolog instance is used to represent the GO feature information of the homologs. The two instances are treated independently to augment the training data. AdaBoost has been used multi-instance learning framework [5], but here we adopt multi-label SVM (support vector machine) as base classifier instead in that SVM is more efficient to handle large data [31].

#### Multi-instance feature construction

Here each protein is represented with two instances, i.e. the target instance and the homolog instance. The homolog instance is constructed using the GO terms of the homologs, which are extracted from SwissProt database [24] using PSI-BLast [32] (E-value = 10) against all species. The GO terms are extracted from GOA database [25]. Using *U* to denote the training set, we obtain two sets of GO terms for each protein *i*, one set denoted as homolog set ${S}_{{}_H}^i$ contains the GO terms of the homologs, and the other set denoted as target set ${S}_{{}_T}^i$ contains the GO terms of the protein itself. Then the set of GO terms of training set *U* is defined as follows:

1 $$ S={\displaystyle \underset{i\in U}{\cup }}\left({S}_{{}_T}^i\cup {S}_{{}_H}^i\right) $$

Based on the denotations, the target instance and the homolog instance are formally defined as follows:

2 $$ {B}_T^i\left[g\right]=\left\{\begin{array}{l}1,g\in {S}_T^i\wedge g\in S\\ {}0,g\notin {S}_T^i\wedge g\in S\end{array}\right.;\kern0.24em {B}_H^i\left[g\right]=\left\{\begin{array}{l}1,g\in {S}_H^i\wedge g\in S\\ {}0,g\notin {S}_H^i\wedge g\in S\end{array}\right. $$

where *B*_*T*_^*i*^[*g*] denotes the component *g* of the target instance *B*_*T*_^*i*^ and *B*_*H*_^*i*^[*g*] denotes the component *g* of the homolog instance *B*_*H*_^*i*^. Formula (2) means that if protein *i* is annotated with the GO term *g*, then the corresponding component in the feature vector *B*_*T*_^*i*^ is set 1; otherwise the component is set 0. Similarly, if the homologs of protein *i* possess the GO term *g*, then the corresponding component in the feature vector *B*_*H*_^*i*^ is set 1; otherwise the component is set 0. If both ${S}_{{}_T}^i$ and ${S}_{{}_H}^i$ are empty set, then protein *i* is removed from the training set.

#### Multi-label learning for modeling cross-talks between signaling pathways

As illustrated in Fig. 1(a), most human signaling pathways share common signaling proteins. From points of view of machine learning, the phenomenon that one protein belongs to multiple signaling pathways is suited to be modeled by multi-label learning. At present there are two approaches to convert multi-label learning into traditional unique-label learning, one approach is label combination method, and the other approach is binary method [33]. Label combination method converts to new label encodings the label combinations that occur in the training data, e.g. the label combination {1, 2, 3} is encoded as {1}, the label combination {1, 4} is encoded as {2}, etc. Binary method trains one binary classifier for each class label by treating as positive the data associated with the class label and treats as negative the data associated with all the other class labels. Here we choose label combination method in that the method trains only one classifier for *n*-class problems, while the binary method needs to train *n* binary classifiers for *n*-class problems.

As compared with traditional supervised learning, the performance estimation of multi-label learning is more complicated. In traditional learning scenario, the standard evaluation criterion is accuracy. In multi-label learning scenario, a direct extension of accuracy is exact match ratio that regards the prediction as correct if and only if all the associated class labels are correctly predicted. However, exact match ratio does not count partial matches that are also significant to expand our knowledge. To take the partial matches into account, we adopt macro-average F-measure and micro-average F-measure [33] as multi-label learning performance metrics. Assume there are *l* testing instances, let *y* ^*i*^ denote the true label vector of the *i*th instance and let *ў* ^*i*^ denote the predicted label vector, then exact match ratio is defined as follows:

3 $$ \frac{1}{l}\sum\limits^{l}_{i=1}I\left[\breve{y}^{i}={y}^{i}\right] $$

where *I* denotes indicator function as defined below:

4 $$ I\left[s\right]=\left\{\begin{array}{l}1s= true\\ {}0s= false\end{array}\right. $$

The above definition of exact match ratio means that the prediction is viewed as correct if and only if all the labels of a protein are correctly recognized. It is easily to see that this definition is too rigorous to take partial matches into account. Actually partial match predictions are also valuable to us. Assuming that a protein is labeled with the label set {1, 2, 4}, the prediction cannot be simply deemed incorrect if the protein is predicted to the label subset {1, 2}, because the partial matches still provide valuable cues to us. For the reason, a proper performance metric for model estimation of multi-label learning should take partial matches into account.

Assume that the total label set is *L* = {1, 2, 3, …, *d*}, for the *i*th instance, the true label set is denoted as *L*_*i*_, and the predicted label set is denoted as *Ĺ*_*i*_. Then a set of *d* binary values are used to formally define the true label and the predicted label for the *i*th instance as follows:

5 $$ {y}^{i}_{j}=\left\{\begin{array}{ll}1&j{\in} L_{i}\\0&j {\notin}L_{i} \end{array},\right. j = 1,2,\ldots,d{\breve{y}}^{i}_{j} = \left\{\begin{array}{ll}1 &j{\in}{\acute{L}}_{i}\\0&j{\notin}{\acute{L}}_{i}\end{array},\right. j=1,2,\ldots,d $$

For label *j*, the performance metric precision (P) and recall (R) are defined as follows:

6 $$ \begin{array}{l}  P= \raisebox{1ex}{$\sum^{l}_{i=1} {\breve{y}}^{i}_{j} {y}^{i}_{j}$}\left/\raisebox{-1ex}{$\sum^{l}_{i=1}{\breve{y}}^{i}_{j}$}\right. ,\\[12pt] R = \raisebox{1ex}{$\sum^{l}_{i=1} {\breve{y}}^{i}_{j} {y}^{i}_{j}$}\left/\raisebox{-1ex}{$\sum^{l}_{i=1}{y}^{i}_{j}$}\right.  \end{array} $$

Since F-measure is defined as $$ F- measure=\raisebox{1ex}{$2\times P\times R$}\!\left/ \!\raisebox{-1ex}{$P+R$}\right. $$, the F-measure for label *j* is formally defined as follows:

7 $$ F\text{-}measure = \raisebox{1ex}{${~}^2{\sum}^{l}_{i=1}\breve{y}_{j}^{i}{y}_{j}^{i}$}\left/ \raisebox{-1ex}{${\sum}^{l}_{i=1}\breve{y}_{j}^{i}+{\sum}^{l}_{i=1}{y}_{j}^{i}$} \right. $$

Macro-average F-measure is defined as the unweighted mean of the F-measures of all class labels:

8 $$ \begin{aligned} & macro\text{-}average\ F\text{-}measure\\ & \ \ \ = \frac{1}{d}\sum\limits^{d}_{j=1}\left(\raisebox{1ex}{${~}^2{\sum}^{l}_{i=1}\breve{y}_{j}^{i}{y}_{j}^{i}$}\left/ \raisebox{-1ex}{${\sum}^{l}_{i=1}\breve{y}_{j}^{i}+{\sum}^{l}_{i=1}{y}_{j}^{i}$} \right.\right) \end{aligned} $$

Micro-average F-measure considers the predictions from all instances and calculates the F-measure across all class labels as follows:

9 $$ \begin{aligned} &micro\text{-}average\ F\text{-}measure\\ &\ \ \ = \raisebox{1ex}{${~}^2{\sum}^{d}_{j=1}{\sum}^{l}_{i=1}\breve{y}_{j}^{i}{y}_{j}^{i}$}\left/ \raisebox{-1ex}{${\sum}^{d}_{j=1}\left({\sum}^{l}_{i=1}\breve{y}_{j}^{i}+{\sum}^{l}_{i=1}{y}_{j}^{i}\right)$} \right. \end{aligned} $$

Both the macro-average F-measure and the micro-average F-measure take partial matches into account. In this work, we use the target instances and the homolog instances separately to estimate the exact match ratio, the macro-average F-measure and the micro-average F-measure. The performance metrics are derived using Gaussian kernel:

10 $$ k\left(x,y\right)= \exp \left(-\gamma \left|\right|x-y\left|\right|{}^2\right) $$

where ||*Δ*|| denotes 2-norm of vector ∆ and the hyperparameter *γ* controls the flexibility of kernel.

## Results

### Performance estimation by 10-fold cross validation

The proposed multi-label multi-instance transfer learning model is estimated by 10-fold cross validation to derive the exact match ratio, macro-average F-measure and micro-average F-measure. In multi-instance learning scenario, each data point is represented with multiple instances, so multiple predicted outcomes are yielded for each test data point in the test or prediction phase. The outcomes are easy to combine into one single outcome in unique-label learning scenario [5]. But outcome combination is not easy in multi-label learning scenario. A proper method is to provide the predicted outcomes of the target instance and the predicted outcomes of the homolog instance. In the training phase, both the target instances and the homolog instances participate in model training.

The computational results are given in Table 2. From Table 2, we can see that the proposed method achieves promising exact match ratio (target instance: 0.7558; homolog instance: 0.7055), which means that over 70 % proteins have their complete label sets correctly recognized. The results are fairly satisfactory though the exact match ratios are moderate, because fully recognizing the complete label set is actually a hard task. The exact match ratio of the homolog instance, though slightly lower than that of the target instance, suggests that the homolog knowledge is useful to the study of novel proteins we know little about. The slight decrease of exact match ratio is partly because the homolog instance carries a certain level of noise that results from evolutionary divergence. When partial matches are taken into account, the proposed model achieves fairly excellent macro-average F-measure (target instance: 0.9555; homolog instance: 0.9267) and micro-average F-measure (target instance: 0.9505; homolog instance: 0.9146). The performance difference between the homolog instance case and the target instance is more subtle, again demonstrating the feasibility of homolog knowledge transfer by means of independent homolog instance.

**Table 2:** Multi-label learning performance estimation by 10-fold cross validation for the target instance case and the homolog instance case

	Exact match ratio	Macro-average F-measure	Micro-average F-measure
Target instance	0.7558	0.9555	0.9505
Homolog instance	0.7055	0.9267	0.9146

**Fig. 2:**
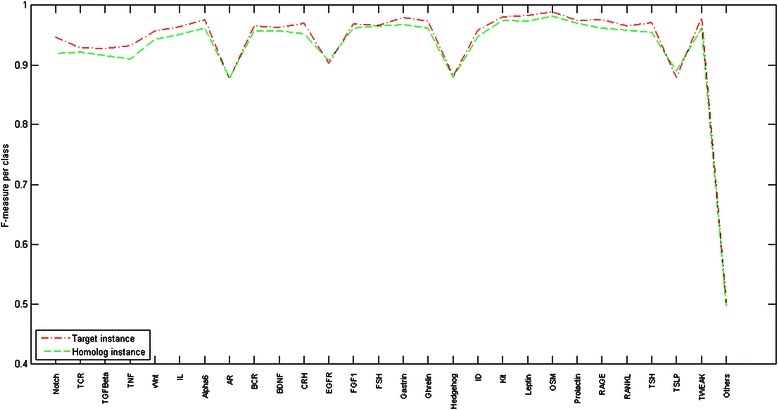
Performance estimation for the 28-class multi-label multi-instance transfer learning model. The F-measure values for each class are illustrated and the curves for the target instance case and the homolog instance case are highly similar

To further estimate the multi-label learning performance, we calculate the F-measure for each class (see Fig. 2). As illustrated in Fig. 2, the proposed method achieves over 0.9 F-measure for most classes. On the four classes (AR, EGFR, Hedgehog, TSLP), the F-measure is between 0.8 and 0.9. On the class *others*, the F-measure is unsatisfactorily about 0.5, partly because of the quality of randomly sampled data. Fortunately, the proposed model achieves sound performances on the 27 human signaling pathways, implying that the misclassifications on the class *others* brings little adverse effect to the 27 signaling pathways. In addition, the performance difference between the homolog instance and the target instance is fairly small (see Fig. 2), suggesting that the predicted outcomes of the homolog instances are equally valuable to us.

### Simultaneous reconstruction of multiple human signaling pathways and their cross-talks modeling

#### Predicting novel signaling components

Recognition of novel signaling components from proteome-wide candidate proteins is the first step of signaling pathway reconstruction. We extract the candidate proteins from SwissProt database [24] and further remove those proteins that have been included in the training data and those proteins that have neither target GO annotations nor homolog GO annotations. Thus we obtain 13,004 candidate proteins in total. The proteome-wide predictions are given in Additional file 1 and the number of the predicted signaling components for each signaling pathway is given in Table 1. The details of the predicted signaling components for each signaling pathway are given in Additional file 2 (target instance) and Additional file 3 (homolog instance). The computational results show that many proteins are predicted to belong to more than one signaling pathway. From Table 1, we can see that the predicted label set of the target instance is much smaller than the predicted label set of the homolog instance and the intersection between the two label sets is not large. The results are largely attributed to the fact that the target instance is generally less enriched in GO annotations while the homolog instance is more enriched in GO annotations but carries a certain level of noise.

#### Linking predicted signaling components to pathways

**Fig. 3:**
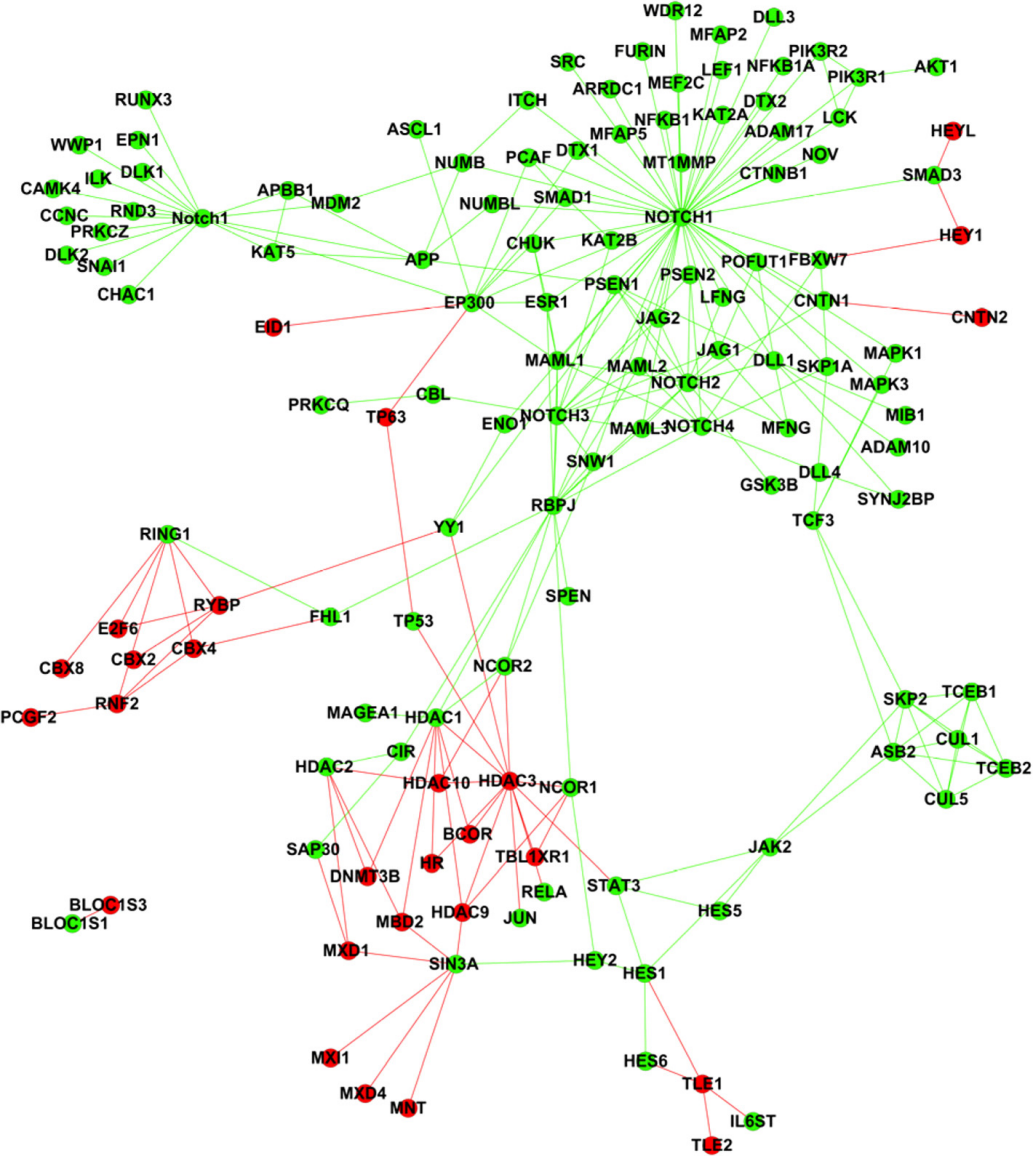
Reconstruction of Notch signaling pathway (target instance). The nodes and edges in green denote the signaling components and signaling PPIs of the experimental Notch signaling pathway. The nodes and edges in red denote the predicted signaling components and the derived signaling PPIs

**Fig. 4:**
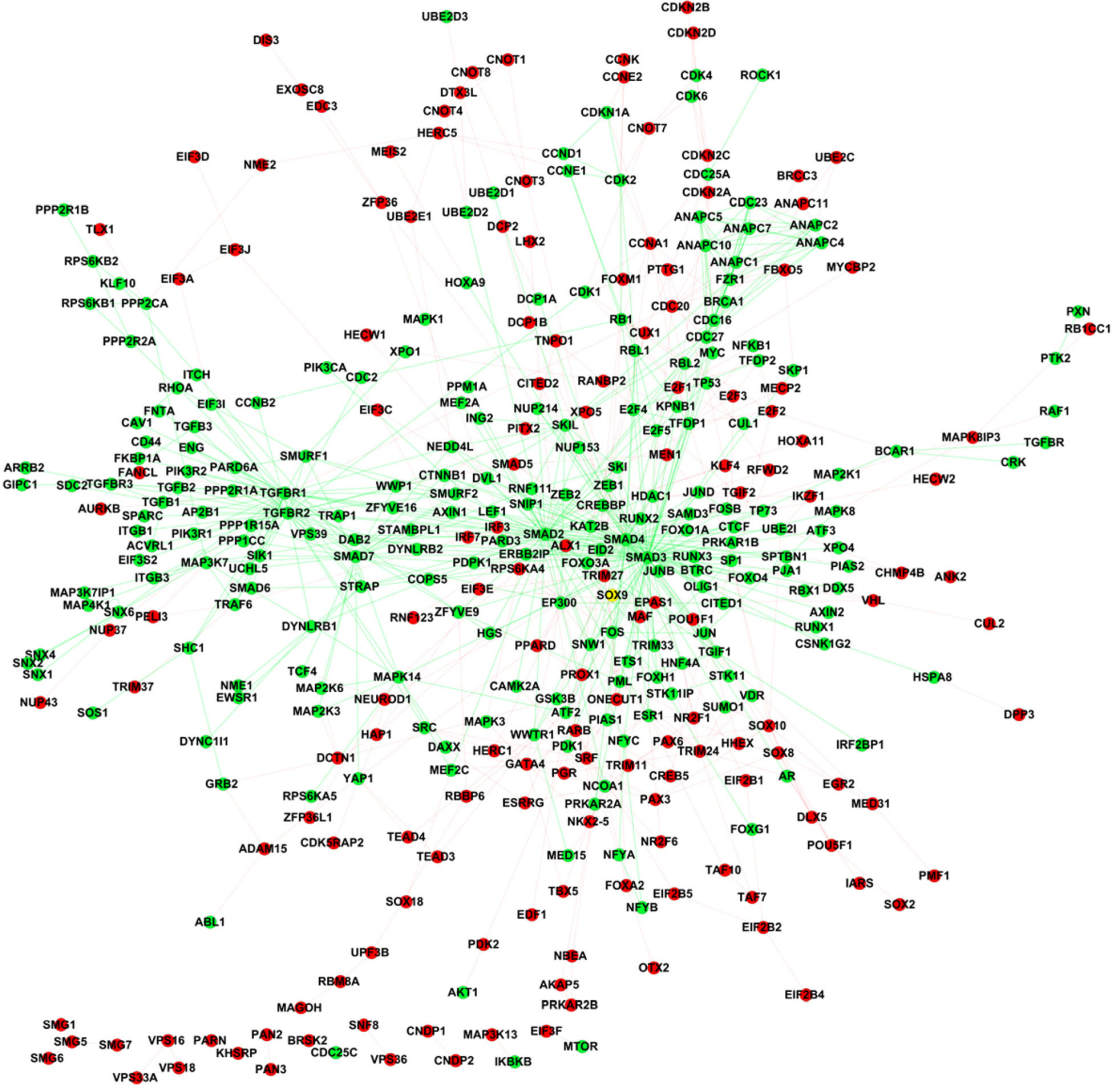
Reconstruction of TGF-βsignaling pathway (target instance). The nodes and edges in green denote the signaling components and signaling PPIs of the experimental Notch signaling pathway. The nodes and edges in red denote the predicted signaling components and the derived signaling PPIs

**Fig. 5:**
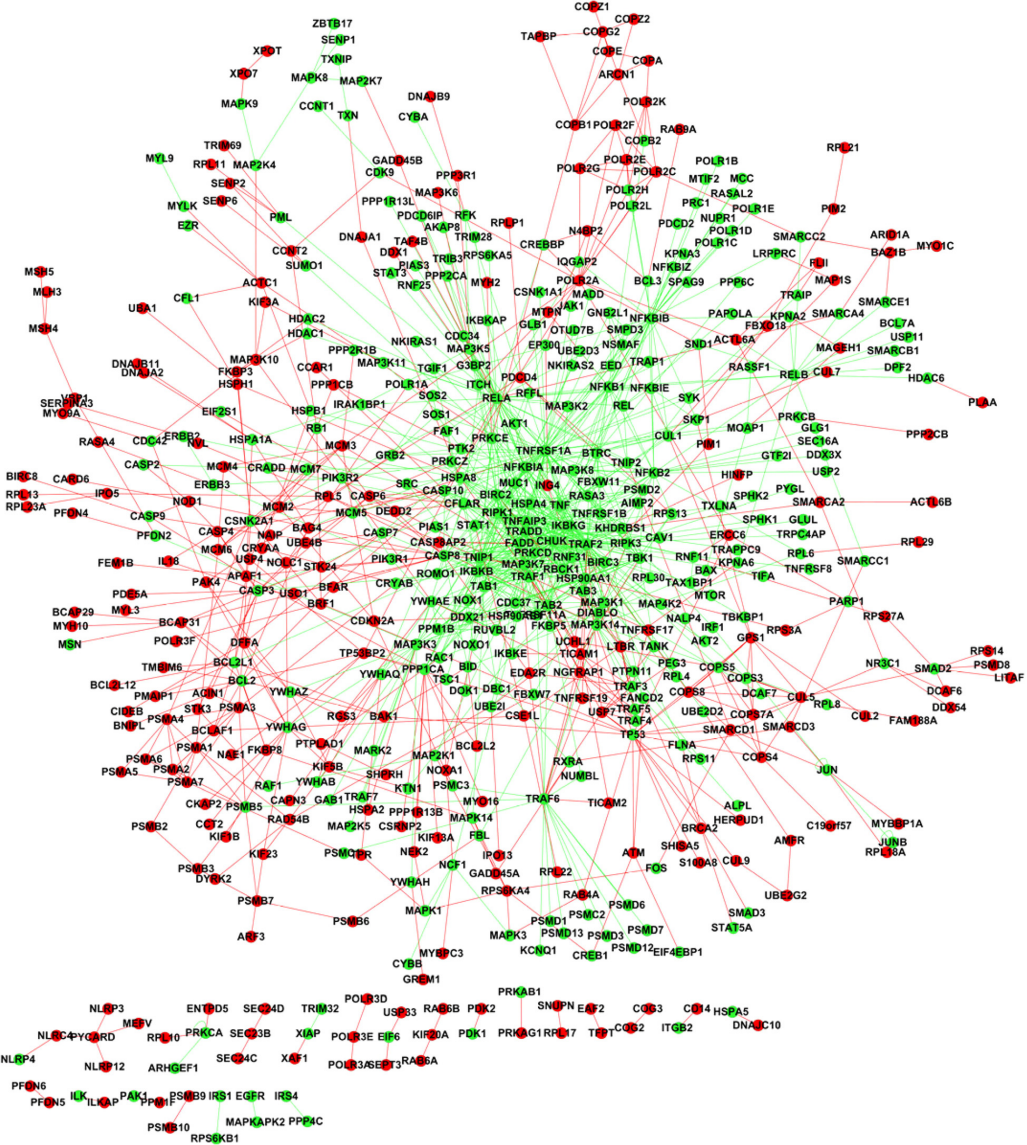
Reconstruction of TNF-αsignaling pathway (target instance). The nodes and edges in green denote the signaling components and signaling PPIs of the experimental Notch signaling pathway. The nodes and edges in red denote the predicted signaling components and the derived signaling PPIs

Signaling proteins generally do not work in isolation but transmit signal via interaction with other proteins or biological molecules. The predicted signaling components needed to be linked to the current human signaling pathways via experimental or predicted protein-protein interactions. For the sake of reliability, we use the experimental PPIs from HPRD database [34] to link the predicted signaling components. Once a predicted signaling component is linked to a signaling pathway, the corresponding PPI becomes a novel signaling PPI of the signaling pathway. Here novel signaling PPI does not mean the PPI is newly predicted, but mean that the PPI is newly treated as a part of the signaling pathway. From HPRD database, we obtain two kinds of signaling PPIs: (1) the PPIs between the predicted signaling components and the known signaling components; (2) the PPIs between the predicted signaling components. The derived signaling PPIs are given in Additional file 4 (target instance) and Additional file 5 (homolog instance). The number of novel signaling PPIs for each signaling pathway is shown in Table 1. Here we link the predicted signaling components to the current signaling pathways via experimental PPIs. We only illustrate Notch, TGF-β and TNF-α signaling pathways that are predicted by the target instances as examples (see Figs. 3, 4 and 5).

As shown in Fig. 3, the predicted signaling components (nodes in red) elongate the existing Notch signaling pathway and form several triangle loops or protein complexes. The signaling pathway is largely elongated at the nodes {RING1, HDAC1, HDAC2, SIN3A, HES1}. At the node RING1, the predicted signaling components form several loops, for instance, a triangle loop {RING1, E2F6, RYBP}. In [35], experimental results demonstrate that E2F6 is a component of the mammalian polycomb complex that interacts with the polycomb group proteins {RYBP, RING1} to play a key role in the regulation of cellular proliferation and terminal differentiation. Centring around RING1, the predicted signaling components {RYBP, E2F6, CBX2, CBX4, CBX8, RNF2, PCGF2} of polycomb complex play important roles in modifying chromatin structure to regulate transcriptional activities, and communicate with the central transcriptional regulator of in Notch signaling via FHL1.

The common topological feature between the extended TGF-β signaling pathway (Fig. 4) and the extended TNF-α signaling pathway (Fig. 5) is that the predicted signaling components generally act as terminal proteins/peripheral proteins, or interact with the peripheral proteins of the existing signaling pathways to form redundant paths or loops. Take the peripheral protein NEDD4L of TGF-β signaling pathway as example (the upper peripheral of Fig. 4), the predicted signaling components {UBE2E1, CNOT4, CNOT8} elongate the TGF-β pathway, wherein CNOT4 has been experimentally demonstrated to activate the JAK/STAT pathway [36]. It can be inferred that CNOT4 acts as a cross-talk signaling component between TGF-β and JAK/STAT signaling pathways.

As illustrated in Fig. 5, the reconstructed TNF-α signaling pathway shows obvious modularity. The predicted signaling components are peripherally distributed to interact with the peripheral proteins of the existing TNF-α signaling pathway, and the interactions between the predicted signaling components elongate the TNF-α pathway with many redundant paths or loops. Take the peripheral protein COPB2 of TNF-α signaling pathway as example (the upper peripheral of Fig. 5), the predicted signaling components {COPA, COPE, COPG2, COPZ2, COPZ1, TAPBP, ARCN1, COPB1} interact with each other and link to the existing signaling component COPB2 via COPA. Moreover, the small motif {COPA, COPE, COPG2, COPZ2, COPZ1, TAPBP, ARCN1, COPB1} also links to the existing signaling component PRKCE (near the core of Fig. 5) via COPB1. The redundant paths help to enhance the robustness of TNF-α signaling pathway. The extended Notch, TGF-βand TNF-α signaling pathways predicted by the homolog instances are given in Additional file 6: Figure S1, Additional file 7: Figure S2 and Additional file 8: Figure S3. Interested readers are referred to Additional files 4 and 5 for other human signaling pathways.

#### Modeling signaling cross-talks

Cross-talk modeling is instrumental to study the regulatory and cooperative relationship between signaling pathways, based on which to further reveal the pathogenesis of diseases [37]. Signaling pathways generally communicate with each other via common signaling components and common signaling PPIs. For simplicity, we investigate here the static map of cross-talks only and do not discuss the temporal and spatial cross-talk mechanism. The details of the predicted common signaling components are given in Additional file 9 (target instance) and Additional file 10 (homolog instance). The experimental signaling components and the predicted signaling components are merged to derive the cross-talk ratio of signaling components *CTR*_*SC*_ as illustrated in Fig. 1(c) (target instance) and Additional file 11: Figure S4 (homolog instance). Comparing Fig. 1(c) and Fig. 1(a), we can see that the cross-talk ratio *CTR*_*SC*_ of the reconstructed signaling pathways is much lower than that of the experimental signaling pathways, in that the predicted novel cross-talk signaling components increase much slower than the predicted novel signaling components. From Fig. 1(c), TCR still significantly correlates with BCR (*CTR*_*SC*_ = 9.2) and EGFR (*CTR*_*SC*_ = 11.5). The details of the common signaling PPIs derived from HPRD database are given in Additional file 12 (target instance) and Additional file 13 (homolog instance). Similarly The experimental signaling PPIs and the predicted signaling PPIs are merged to derive the cross-talk ratio of signaling PPIs *CTR*_*SPPI*_ as illustrated in Fig. 1(d) (target instance) and Additional file 14: Figure S5 (homolog instance).

**Fig. 6:**
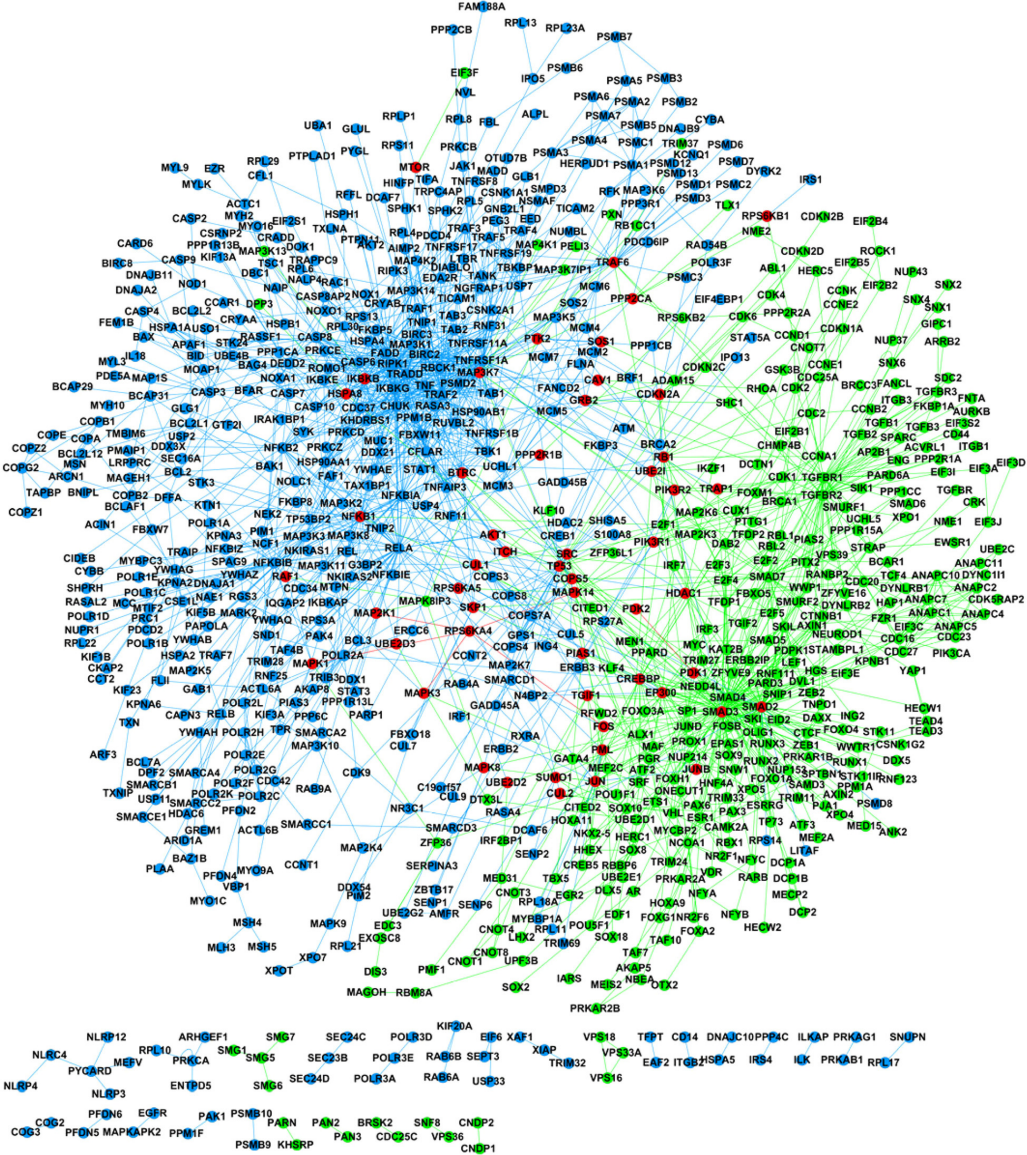
Cross-talks between TGF-βsignaling pathway and TNF-αsignaling pathway (target instance). The nodes and edges in green denote the predicted signaling components and derived signaling PPIs of TGF-βsignaling pathway. The nodes and edges in blue denote the predicted signaling components and derived signaling PPIs of TNF-αsignaling pathway. The nodes and edges in red denote the common signaling components and the common signaling PPIs

The static map of cross-talks between TGF-β signaling pathway and TNF-αsignaling pathway (target instance) is illustrated in Fig. 6, where the color green denotes TGF-βsignaling components and signaling PPIs, the color blue denotes TNF-α signaling components and signaling PPIs, and the color red denotes the cross-talk signaling components and the cross-talk signaling PPIs. There are 52 cross-talk signaling components and 6 cross-talk signaling PPIs between TGF-β signaling pathway and TNF-α signaling pathway, of which 6 cross-talk signaling components and the 6 cross-talk signaling PPIs are predicted. From Fig. 6, we can see that most of the cross-talk signaling components are peripheral proteins at the cross boundaries of the two signaling pathways except several hub proteins (e.g. TGF-β: SMAD2, SMAD3, JUNB; TNF-α: MAP3K, HSPA8, IKBKB, etc.).

#### Literature and KEGG validation

We further validate the proteome-wide predictions against recent literature and signaling pathway databases. Since the data we are concerned about are scarce and sparsely scattered among hundreds of literature, it is hard to collect sufficient evidences to validate the predictions. Nevertheless, we still find dozens of supporting evidences as shown in Table 3. For instances, four evidences are found for Notch signaling pathway. For the predicted signaling components or targets {RNF2, RING1B}, [38] has experimentally demonstrated that the Polycomb protein Ring1B promote the proliferation and self-renewal of embryonic neural stem/progenitor cells by repressing cell cycle inhibitors and maintaining Notch signaling pathway. For the predicted signaling component TBL1XR1, [39] has experimentally demonstrated that TBL1XR1 acts as a key player in the regulation of multiple signaling pathways (Wnt/β-catenin, Notch, NF-κB, and nuclear receptor) and gene transcription. For POGLUT1, [40] has demonstrated that POGLUT1 is a part of Notch signaling pathway that encodes protein O-glucosyltransferase 1 and is involved in posttranslational modification of Notch proteins. For the predicted signaling component SNX27 of TCR signaling pathway, [41] has experimentally shown that SNX27 is identified as a PDZ-containing component of the T cell immunological synapse and SNX27-positive endosomes polarise to the immunological synapse in response to TCR activation. As for TGF-βsignaling pathway, the proteins {RBPMS, BMP15} are predicted to be singnaling components. [42] shows that RBPMS interacts with TGF-β receptor type I (TbR-I), increases phosphorylation of C-terminal SSXS regions in Smad2 and Smad3, and promotes the nuclear accumulation of the Smad proteins. Fenwick 2013 [43] shows that BMP15 is a closely related TGF-βligand that is implicated as key regulators of follicle development and fertility. As for TNF-αsignaling pathway, [44] experimentally demonstrates that knock-down of the TNFα-induced protein TNFAIP8 in tumor cells decreases their oncogenicity, which suggests TNFAIP8 may be involved in carcinogenesis. As for WNT signaling pathway, [45] shows that the interaction between Hhex and SOX13 modulates Wnt/TCF pathway activity, and the interaction between SOX13 and TCF1 represses Wnt/TCF signaling. As for BCR signaling pathway, the Ingenuity Pathways Analysis shows that MAP3K12 is involved in BCR signaling pathway and PIK3C3 is involved in Prolactin signaling pathway [46]. As for Hedgehog signaling pathway, [47] has experimentally demonstrated that MED12 is linked biochemically and genetically to Hedgehog signaling pathway.

**Table 3:** Validation of the predicted signaling components against recent literature and KEGG database

Pathway	KEGG		Literature
	Target instance	Homolog instance	
Notch	{RFNG}	{DTX4,RFNG,KAT2A,DTX2,DTX3L}	{RNF2,RING1B} [38]; {TBL1XR1} [39];{POGLUT1} [40]
TCR	{CD8B,PAK2}	{PAK7,PAK2,IFNG,PAK4,AKT3}	{SNX27} [41]
TGFBeta	{SMAD5,CDKN2B,PITX2}	{GDF5,AMHR2,BMP5,PPP2CB,GDF7,ACVR1B,PITX2,INHBA,BMPR2,SMAD5,GDF6,ACVR2A,ACVR2B,INHBB,BMPR1A,ACVR1,BMP7,BMPR1B}	{RBPMS} [42]; {BMP15} [43]
TNF	{BAG4,RPS6KA4}	{CREB3L3,LTA}	{TNFAIP8} [44]
Wnt	{WNT16,WNT8A,WNT2B,DKK4,CSNK1A1L,WNT8B,GPC4,CXXC4,SFRP5,CTNNBIP1,NKD2,PORCN,TCF7L1,NKD1,SOX17,CSNK2A2,WNT10B,DKK2,APC2,TCF7,WNT10A}	{WNT16,PPARD,WNT8A,WNT2B,DKK4,WNT8B,CSNK1A1L,GPC4,CXXC4,PPP3CC,SFRP5,CTNNBIP1,PORCN,TCF7L1,CSNK2A2,WNT10B,DKK2,APC2,TCF7,WNT10A}	{WNT9A,SOX13} [45];
BCR		{AKT3,LILRB3}	{MAP3K12} [46]
Hedgehog		{ZIC2}	{MED12} [47]
Prolactin		{AKT3}	{PIK3C3} [46]

The evidences that support the proteome-wide predictions are very limited, so we resort to KEGG database [19] for further validation. Although the data in KEGG database are not newly published or updated, the data that are collected in KEGG database but not collected in NetPath database are also suited to be used as validation data. At present, the overlap rate of signaling components between NetPath and KEGG is very low. For instances, the overlap rate of TGF-βsignaling pathways between the two databases is 22.62 % and the overlap rate of TNFαsignaling pathways is only 13.77 %. Here more than sixty predicted signaling components are validated against KEGG database (see Table 3), suggesting that the proteome-wide predictions yielded by the proposed method are reliable. From Table 3, we can see that the homolog instances recognize more novel signaling components than the target instances, once again demonstrating that the homolog instances also yield valuable predictions.

## Discussion

Signaling pathways play significant roles in the biological processes of cell growth, cell differentiation, cell apoptosis and organism development. At present, the current signaling pathways are far from complete. Computational modeling helps to accelerate the proteome-wide reconstruction and global cross-talk mapping of human signaling pathways. The existing computational methods focus on predicting signaling components and/or deriving orthologous signaling PPIs from the topology of signal transduction networks, or describing the molecular dynamics of signaling pathways. To our knowledge, no computational methods have been reported to simultaneously take more than two signaling pathways into account and explicitly predict their cross-talks. In this work, we propose a multi-label multi-instance transfer learning method to simultaneously reconstruct 27 human signaling pathways and model their cross-talks. The known signaling components of 27 human signaling pathways are directly exploited to train a 28-class predictive model (the 28th class is the negative class) and the model is used to predict proteome-wide novel signaling components. Then the predicted signaling components are linked to the current signaling pathways via the experimental PPIs in HPRD database. Based on the predicted signaling components and the derived signaling PPIs, we can conveniently reconstruct the 27 human signaling pathways and derive their cross-talks. Computational results show that both the target instances and the homolog instances achieve satisfactory multi-label learning performance and the homolog instances also yield valuable predictions. Some of the proteome-wide predictions have been validated against recent literature and KEGG database.

### Gene ontology enrichment analysis

We conduct gene ontology enrichment analysis of the predicted signaling components to get knowledge about the biological processes that the signaling pathways are involved in. Take TGF-βsignaling pathway and TNF-αsignaling pathway (predicted by the target instances)for examples, 27.4 % predicted TGF-βsignaling components are annotated with the term GO:0006355 (regulation of transcription, DNA-dependent), 15.75 % predicted TGF-βsignaling components are annotated with the term GO:0016567 (protein ubiquitination) and 11.30 % predicted TGF-βsignaling components are annotated with the term GO:0007275 (multicellular organismal development). As regards with the predicted TNF-αsignaling components, the GO enrichment for GO:0006915 (apoptotic process), GO:0006457 (protein folding) and GO:0006954 (inflammatory response) are 13.63, 4.29 and 3.98 %, respectively.

**Fig. 7:**
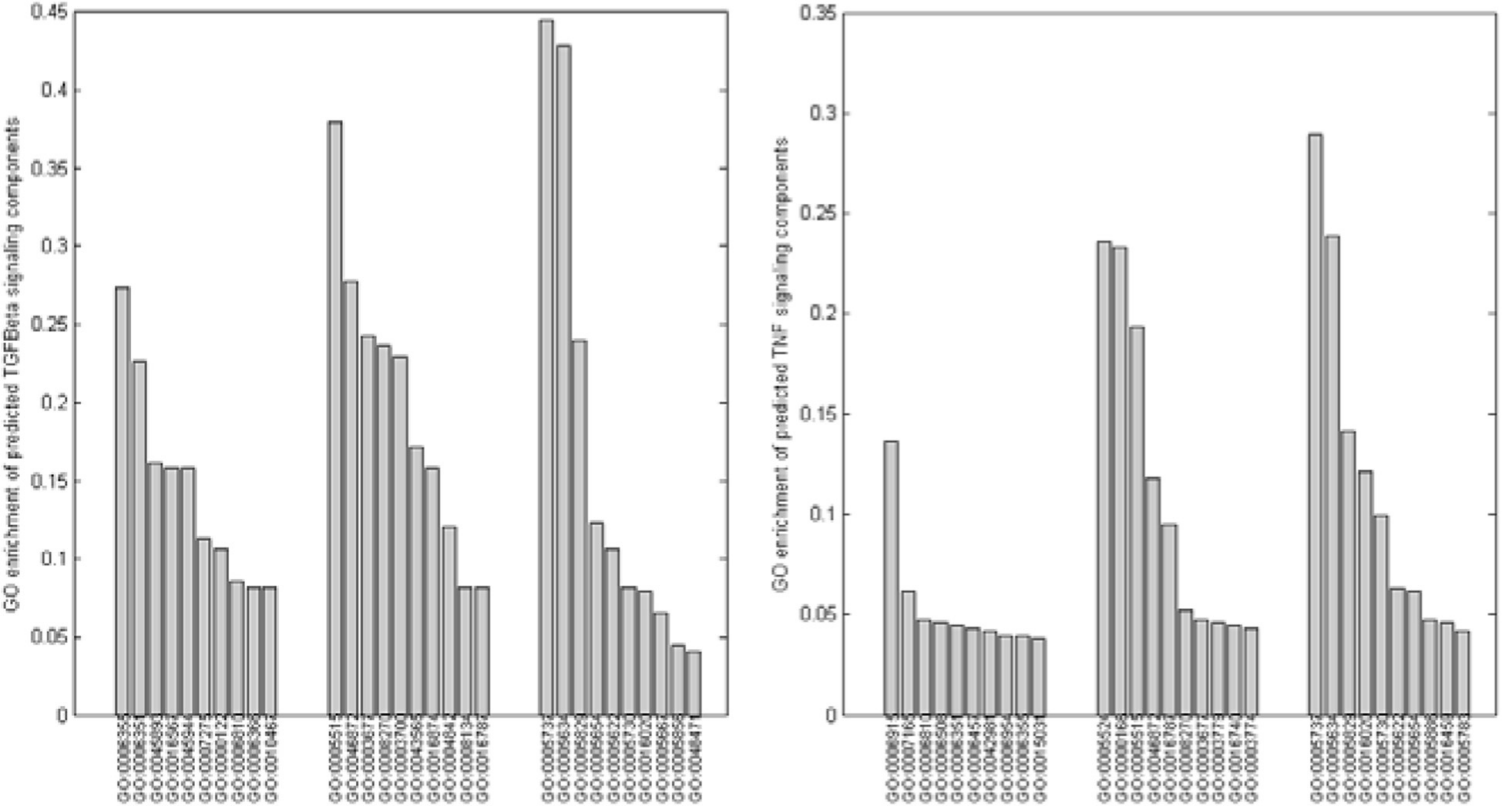
GO enrichment analysis of the predicted signaling components for TGF-β(left pane) and TNF-α(right pane) signaling pathways. For each pane, three groups of GO enrichment analysis are shown (left: biological processes; middle: molecular functions; right: cellular components). For each group of GO terms, only 10 top GO enrichments are given

Next we study the molecular functions that the predicted signaling components fulfil. As for TGF-βsignaling pathway, the GO enrichment of the terms GO:0005515 (protein binding), GO:0046872 (metal ion binding) and GO:0004842 (ubiquitin-protein ligase activity) are 38.01, 27.74 and 11.99 %, respectively. As for TNF-αsignaling pathway, the GO enrichment for GO:0005524 (ATP binding), GO:0005515 (protein binding) and GO:0046872 (metal ion binding) are 23.58, 19.30 and 11.79 %, respectively. As for the cellular compartments that the predicted signaling components reside in, a majority of the predicted TGF-βand TNF-αsignaling components are located in cytoplasm (GO:0005737), nucleus (GO:0005634) and cytosol (GO:0005829). The GO enrichment analysis of predicted TGF-βand TNF-αsignaling components is illustrated in Fig. 7, where only 10 top GO enrichments are given for each aspects of gene ontology. The full GO enrichment analysis of the predicted signaling components are given in Additional file 15 (biological processes), Additional file 16 (molecular functions) ad Additional file 17 (cellular compartments).

### Validation against Reactome database and Signalink database

Apart from NetPath and KEGG, the other signaling pathway databases such as Reactome [20] and Signalink [21, 23] have also curated several human cancer signaling pathways. Among the databases, Reactome is most frequently updated and is larger than or equivalent to NetPath in terms of the size of human cancer signaling pathways. For instances, in Reactome the number of signaling components of {Notch, TGFBeta, Wnt} signaling pathways is {111, 72, 294}, respectively. In Netpath, the number of signaling components of {Notch, TGFBeta, Wnt} signaling pathways is {126, 220, 120}, respectively (the sizes of the training data of {Notch, TGFBeta, Wnt} are 83, 216 and 108 after removing those hypothetical/unreviewed and unannotated proteins). In Signalink, the number of signaling components of {Notch, TGFBeta, Wnt} signaling pathways is {21, 73, 95}, respectively. In this work, we adopt NetPath because it curates the largest number of human cancer signaling pathways and the sizes of the signaling pathways are moderate. To further validate the reliability of the proposed method, we also validate the proteome-wide predictions against Reactome and Signalink (see Table 4). The validation data from Reactome and Signalink are not contained in the training data. From Tables 4 and 3, we can see that Reactome validates much more predictions than KEGG and Signalink partly because it is timely updated. Take TCR signaling pathway for instance, Reactome validates 24 predictions (target instance) and 32 predictions (homolog instance), much larger than KEGG (2 target-instance predictions and 5 homolog-instance predictions). With more experimental discoveries are made, more proteome-wide predictions are supposed to be validated.

**Table 4:** Validation of the predicted signaling components against Reactome database and Signalink database

Pathway	Reactome		Signalink	
	Target instance	Homolog instance	Target instance	Homolog instance
Notch	{HDAC9,POGLUT1,HEYL,TLE2,TBL1XR1,TLE4,TLE1,HEY1,TLE3,B4GALT1,DLK1,CCNC,HDAC3,RFNG,HDAC10}	{TBL1XR1,HDAC4,DTX4,RFNG,POGLUT1,HEYL,HDAC9,KAT2A,HDAC8,DTX2,HEY1,DLK1,MIB2,HDAC10,HDAC3}		
TCR	{MRC1,KLC1,TUBA1C,PAK2,TUBB3,KLRK1,KLRG1,CD8B,TUBA4A,TUBB4A,TUBB4B,TUBB2B,PDGFRA,DCTN2,TUBA1B,CD226,LILRB2,ITGAL,TUBB2A,TUBB6,KIF15,TUBB1,KIF5A,LILRB3}	{RNF138,PAK2,TUBB3,ERAP1,CD274,OSBPL1A,KIR2DL1,AGO3,TUBA1B,AKT3,TUBB6,TUBB2A,TUBA3C,ANAPC11,RNF41,TUBA1C,KIR3DL1,DNM3,TUBA4A,SPTBN2,PHLPP1,TUBB4B,TUBB4A,TUBB2B,TRIM21,CXADR,RNF123,TRIM9,PVRL2,TRIM11,TUBB1,PHLPP2}	-	
TGFBeta	{MEN1,UBB,TGIF2,CDKN2B}	{UBE2M,TGIF2,RPS27A,MTMR4}	{MTMR4,WWOX,GDF6,ACVR2B,ACVR1B,ACVR1,BMP7,INHBA,BMPR2,BMPR1B}	
Wnt	{TNKS2,WNT16,WNT8A,PYGO2,WNT2B,DKK4,WNT8B,CXXC4,RSPO4,AMER1,CTNNBIP1,PORCN,RNF43,TCF7L1,KREMEN2,SOX17,DACT1,CSNK2A2,WLS,WNT10B,DKK2,CCDC88C,TCF7,PYGO1,WNT10A}	{WNT16,PPP2CB,WNT8A,WNT2B,WNT8B,AMER1,CTNNBIP1,DACT1,CSNK2A2,SOX13,PYGO2,DKK4,CXXC4,PORCN,TCF7L1,KREMEN2,SOX6,BCL9L,WLS,WNT10B,DKK2,TCF7,WNT10A}	{WNT8A,APC2}	
BCR	{ITPR3}	{AKT3,ITPR3}	-	
EGFR	{AGO4,AP2A2,MLST8,FGF3,AGO3,AKT3,RPS27A}	{AP2A2,PDGFRA,AKT3,ADAM12,PDGFB,PHLPP2}	-	

The sign “-” denotes that Signalink does not curate the signaling pathway

The quality of signaling PPIs largely depends on the quality of human PPI database. Here we adopt HPRD database [34] (http://www.hprd.org/) for primary research since HPRD focuses on collecting reliable protein-protein interactions of Homo sapiens. However, HPRD is not so frequently updated as Reactome. In the future work, we will combine the updated PPI databases (HPRD, Reactome, Signalink, etc.) with computational PPI predictions to update the reconstructed signaling pathways. Fortunately, the predicted signaling components are very conveniently linked to signaling pathways via newly derived PPIs.

### Comparison with the existing methods

The existing computational methods for reconstruction of signaling pathways are largely classified into two categories: graph search methods [9–11] and machine learning methods [14–16]. Graph search methods rely on PPI network topology to search for signaling pathways. These methods are simple with least data constraints, but feedback loops make the shortest path algorithm inaccurate. The existing machine learning methods focus on the discovery of novel signaling components. These methods exploit the experimental data of signaling components and mainly predict orthologues signaling pathways, but the methods seldom simultaneously exploit more than two signaling pathways and model their cross-talks. The proposed multi-label multi-instance method simultaneously exploits 27 human cancer signaling pathways to model the phenomenon that a signaling protein belongs to more than two signaling pathways. As compared with the existing methods, our method has the merit of explicit knowledge sharing and knowledge transfer between signaling pathways. After linking the predicted signaling components to signaling pathways, we can easily derive the cross-talk signaling components and cross-talk signaling PPIs.

### Applicability

The method can be extended to solve other biological problems. The computational results provided in the supplementary files can be used as benchmark for novel method development or be used for further biomedical research.

## Conclusion

In this work, we propose a multi-label multi-instance method to simultaneously reconstruct 27 human cancer signaling pathways and model their cross-talks. The proposed method demonstrates satisfactory multi-label learning performance and some of the proteome-wide predictions are validated against the signaling pathway databases (KEGG, Reactome and Signalink) and recent literature. The method and the results can be used for further model development and further biomedical research.

## Additional files

Additional file 1:
**Text file contains the total predicted signaling components.** (ZIP 203 KB)
Additional file 2:
**Text file contains the predicted signaling components for each human signaling pathway (target instance).** (ZIP 37 KB)
Additional file 3:
**Text file contains the predicted signaling components for each human signaling pathway (homolog instance).** (ZIP 49 KB)
Additional file 4:
**Text file contains the derived signaling PPIs for each human signaling pathway (target instance).** (ZIP 17 KB)
Additional file 5:
**Text file contains the derived signaling PPIs for each human signaling pathway (homolog instance).** (ZIP 23 KB)
Additional file 6: Figure S1. Reconstructed Notch signaling pathway (homolog instance). (JPG 430 KB)
Additional file 7: Figure S2. Reconstructed TGF-βsignaling pathway (homolog instance). (JPG 991 KB)
Additional file 8: Figure S3. Reconstructed TNF-αsignaling pathway (homolog instance). (JPG 1254 KB)
Additional file 9:
**Text file contains the predicted cross-talk signaling components between human signaling pathways (target instance).** (ZIP 4 KB)
Additional file 10:
**Text file contains the predicted cross-talk signaling components between human signaling pathways (homolog instance).** (ZIP 36 KB)
Additional file 11: Figure S4. The cross-talk ratio of signaling components *CTR*_*SC*_ of the reconstructed human signaling pathways (homolog instance). (JPG 168 KB)
Additional file 12:
**Text file contains the derived cross-talk signaling PPIs between human signaling pathways (target instance).** (ZIP 17 KB)
Additional file 13:
**Text file contains the derived cross-talk signaling PPIs between human signaling pathways (homolog instance).** (ZIP 23 KB)
Additional file 14: Figure S5. The cross-talk ratio of signaling PPIs *CTR*_*SPPI*_ of the reconstructed human signaling pathways (homolog instance). (JPG 173 KB)
Additional file 15:
**Text file contains the GO enrichment analysis of the predicted signaling components (biological processes).** (ZIP 702 KB)
Additional file 16:
**Text file contains the GO enrichment analysis of the predicted signaling components (molecular functions).** (ZIP 143 KB)
Additional file 17:
**Text file contains the GO enrichment analysis of the predicted signaling components (cellular compartments).** (ZIP 171 KB)
