# Supplementary figures and images for: Multi-label multi-instance transfer learning for simultaneous reconstruction and cross-talk modeling of multiple human signaling pathways
Source: BMC Bioinformatics. 2015 Dec 30;16:417. doi: 10.1186/s12859-015-0841-4 (PMC4697333; doi:10.1186/s12859-015-0841-4)

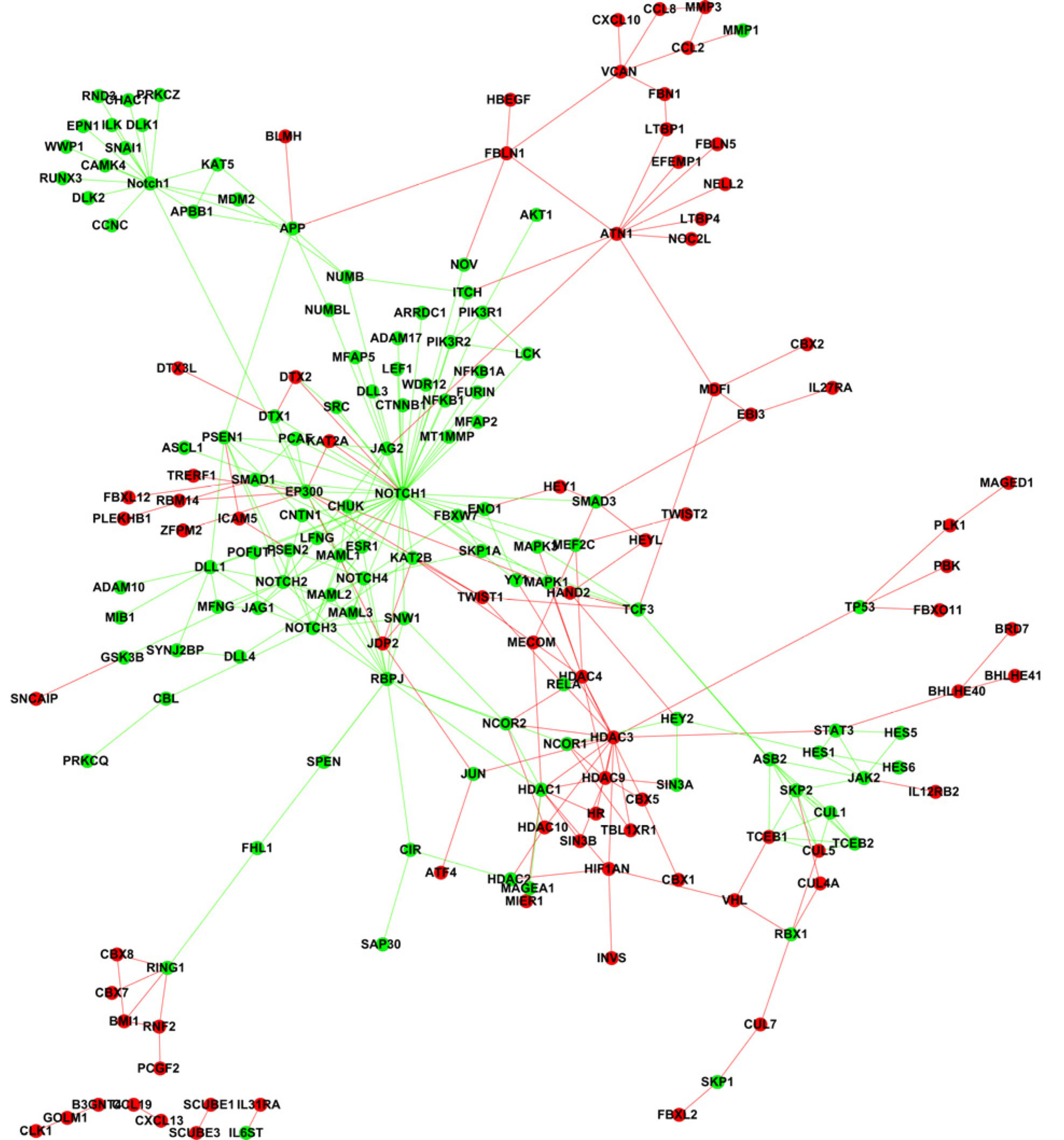

Supplement: Additional file 6: Figure S1. — Reconstructed Notch signaling pathway (homolog instance). (JPG 430 KB) [file 12859_2015_841_MOESM6_ESM.jpg]

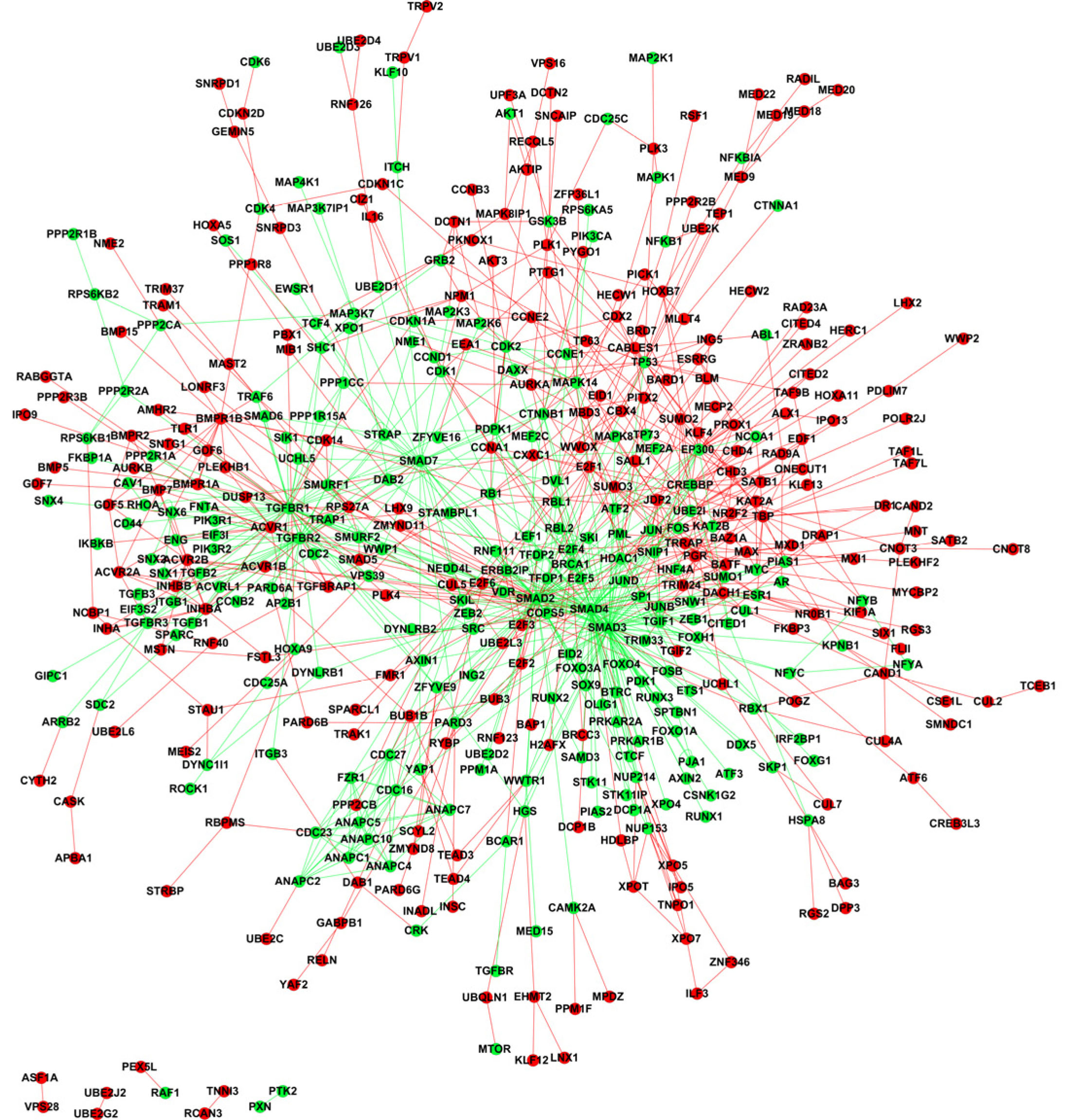

Supplement: Additional file 7: Figure S2. — Reconstructed TGF-βsignaling pathway (homolog instance). (JPG 991 KB) [file 12859_2015_841_MOESM7_ESM.jpg]

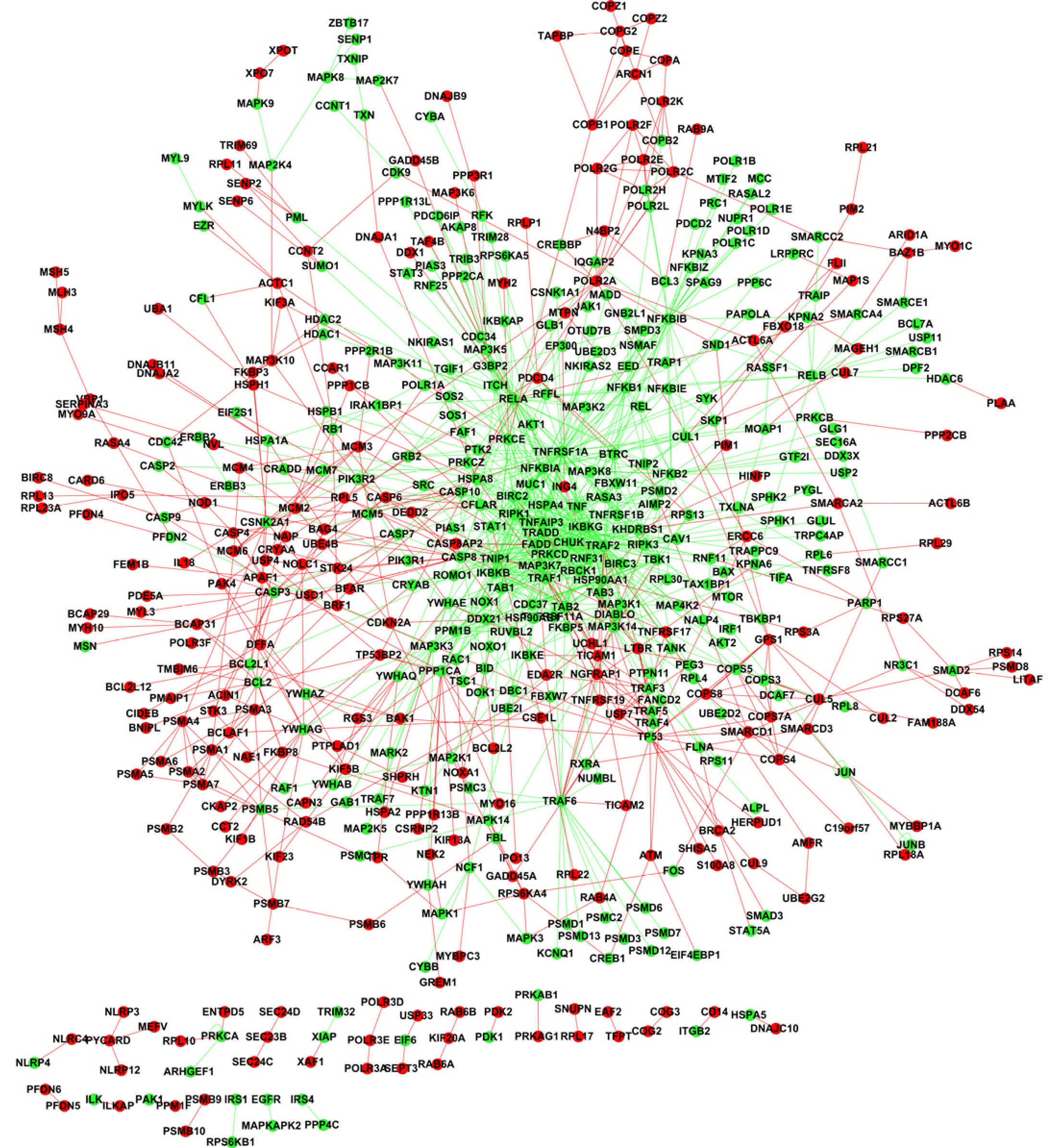

Supplement: Additional file 8: Figure S3. — Reconstructed TNF-αsignaling pathway (homolog instance). (JPG 1254 KB) [file 12859_2015_841_MOESM8_ESM.jpg]

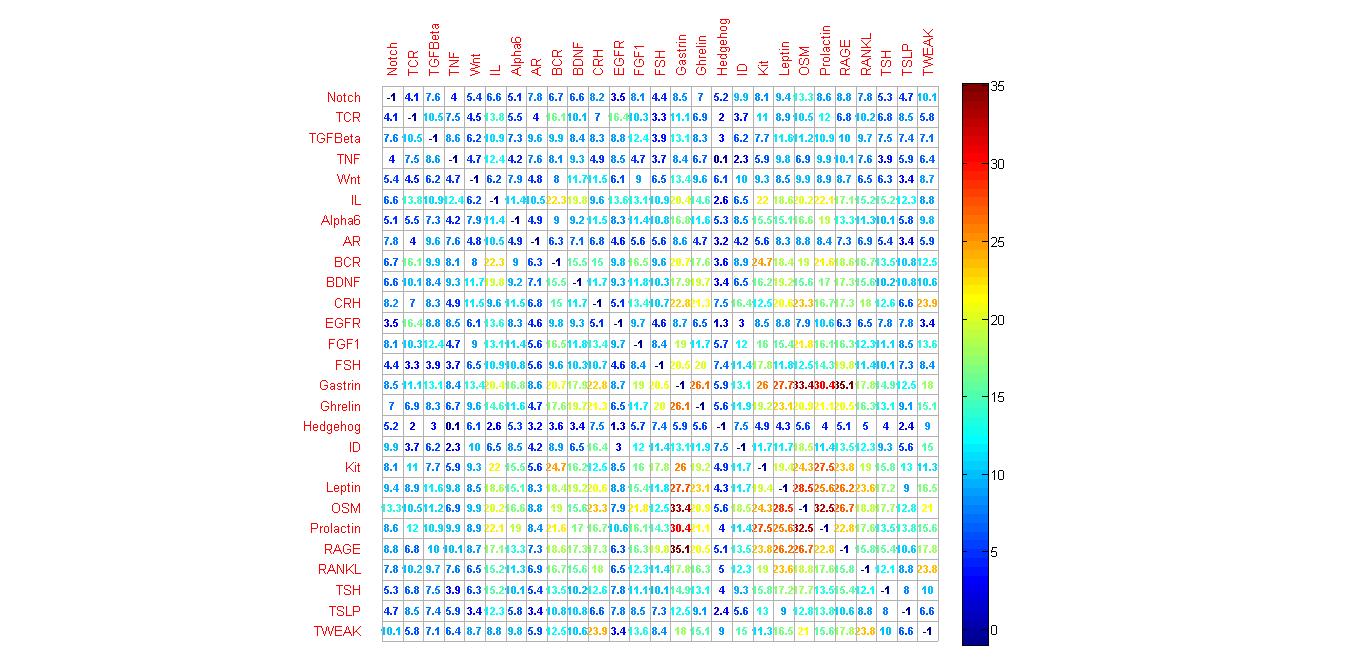

Supplement: Additional file 11: Figure S4. — The cross-talk ratio of signaling components CTR SC of the reconstructed human signaling pathways (homolog instance). (JPG 168 KB) [file 12859_2015_841_MOESM11_ESM.jpg]

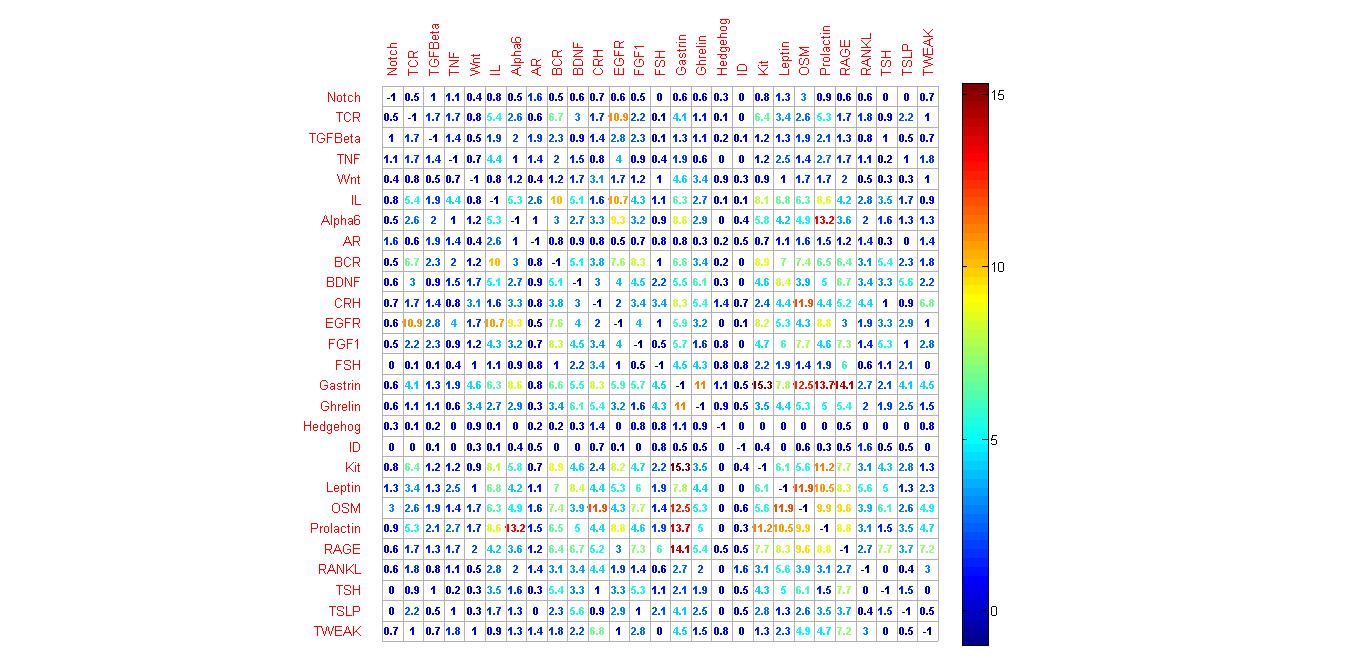

Supplement: Additional file 14: Figure S5. — The cross-talk ratio of signaling PPIs CTR SPPI of the reconstructed human signaling pathways (homolog instance). (JPG 173 KB) [file 12859_2015_841_MOESM14_ESM.jpg]
